# Effect of tesamorelin in people with HIV with and without dorsocervical fat: Post hoc analysis of phase III double-blind placebo-controlled trial

**DOI:** 10.1017/cts.2022.515

**Published:** 2022-12-07

**Authors:** Farah Rahman, Taryn McLaughlin, Pedro Mesquita, Josee Morin, Diane Potvin, Marilyn De Chantal, Judith A. Aberg

**Affiliations:** 1 Department of Medicine, Division of Infectious Diseases, Icahn School of Medicine at Mount Sinai, New York, NY, USA; 2 Theratechnologies Inc., Montréal, Canada; 3 Innovaderm Research, Montréal, Canada

**Keywords:** HIV, antiretrovirals, lipohypertrophy, growth hormone-releasing hormone, tesamorelin

## Abstract

Tesamorelin, a synthetic growth hormone-releasing hormone, is indicated for the reduction of visceral adipose tissue (VAT) in people with HIV. Here, we performed a post hoc analysis of participants receiving tesamorelin for 26 weeks in a phase III clinical trial. Efficacy data were compared between individuals with and without dorsocervical fat, stratified by tesamorelin response. Among tesamorelin responders, VAT and waist circumference (WC) decreased in both dorsocervical fat groups and did not statistically differ (VAT *P* = 0.657, WC *P* = 0.093). These data demonstrate that tesamorelin is equally effective and should be considered in the treatment of excess VAT regardless of the presence of dorsocervical fat.

## Introduction

Lipodystrophy is a spectrum of fat redistribution and metabolic disorders, including lipoatrophy and lipohypertrophy. Lipoatrophy is characterized by loss of subcutaneous fat, usually in the limbs, face, or buttocks. Lipohypertrophy is characterized by fat deposition in abdominal visceral adipose tissue (VAT), breasts, and/or dorsocervical area [1]. It is a known manifestation among people with HIV (PWH) treated with antiretroviral therapy (ART) leading to the term HIV-associated lipodystrophy. This term, however, is an oversimplification, as it varies between patients. Some may present with lipohypertrophy or lipoatrophy or a combination of both [1].

Multiple factors contribute to lipodystrophy in PWH, including patient characteristics, HIV viral load, nadir CD4 count, and duration or type of ART [2]. Impaired fatty acid metabolism and endocrine alterations, including adiponectin deficiency, abnormal leptin regulation, and and impaired growth hormone (GH) secretion, have also been associated with this condition [2]. Specifically, GH serum levels have been found to be significantly lower in persons with lipodystrophy regardless of HIV status. Furthermore, there is an inverse correlation between VAT and mean overnight GH exposure [3].

Beyond traditional HIV-associated lipodystrophy, VAT has been identified as a component of ART-associated weight gain [4]. This weight gain following ART initiation was initially attributed to the “return to health” phenomenon related to immunologic recovery [5]. Weight gain, however, has also been observed in individuals on stable antiretroviral regimens switching to newer classes of drugs [6]. Indeed, recent studies have shown weight gain associated with modern ART, specifically integrase inhibitors, such as dolutegravir (DTG) and tenofovir alafenamide (TAF) [7]. Consistent with these findings, the ADVANCE study, an open-label, 96-week study in South Africa, showed more weight gain in the arms containing TAF and/or DTG than the arm without [8]. Furthermore, this weight gain was associated with a VAT increase [9]. Similarly, VAT increases were observed following initiation with ART in a US-based study evaluating regimens anchored on integrase inhibitors, protease inhibitors, and non-nucleoside reverse transcriptase inhibitors [10]. Thus, VAT increases have been observed across a variety of ART regimens and clinical settings.

There are treatments for lipodystrophy, including tesamorelin, a synthetic growth hormone-releasing hormone (GHRH) approved by the Food and Drug Administration (FDA) for reduction of VAT [11]. Tesamorelin has been shown to reduce VAT quantity and improve VAT quality [12,13]. There is no data, however, on the impact of tesamorelin on VAT in patients with different presentations of lipodystrophy, such as with excess dorsocervical fat. After a commercial insurer declined authorization for the use of tesamorelin in a patient with VAT and dorsocervical fat, we aimed to assess if tesamorelin would have similar efficacy profiles among PWH with and without dorsocervical fat.

## Materials and Methods

Post hoc analysis was done on Theratechnologies’s phase 3 randomized, double-blind, multicenter trials. The study was approved by Institutional Review Boards at each site and informed consent was obtained from all individual participants included in this study. Patients, were eligible for the study if they were between 18 and 65 years of age, had confirmed HIV infection and had evidence of VAT accumulation, defined as waist circumference (WC) > 95 cm and waist-to-hip ratio (WHR) > 0.94 for males, and WC > 94 cm and WHR > 0.88 for females. Participants were also required to have been on stable ART regimen for 8 weeks or more. Dorsocervical status of participants was measured at baseline, prior to tesamorelin administration. This was based on both patient and physician assessment of patient’s dorsocervical fat deposition and was reported as absent or present. Participants were randomized to receive tesamorelin 2 mg or placebo daily for 26 weeks (main phase). At the end of the main phase, participants on the placebo arm were reassigned to tesamorelin and those on tesamorelin were re-randomized to either remain in that arm or cross-over to placebo until 52 weeks. All study staff were blinded to all assignments. Body composition, biochemical testing, and body image parameters were determined as previously described [12]. Data points were collected at weeks 26 and 52; however, the analysis in this study is limited to week 26. For this post hoc analysis, placebo participants were not included in the statistical analysis as the main interest was in patients receiving tesamorelin. A per-protocol analysis was used in order to analyze the effects of tesamorelin in patients who were adherent to treatment. Tesamorelin responders were defined as patients with at least 8% decrease in VAT as determined by CT scan, who were adherent to medication (> 80% compliance), had no major protocol violation and underwent ≥ 1 post-dose abdominal computed tomography. Percentage VAT change was calculated as the change between baseline and 26 weeks. Results are reported for two sets of participants – those who responded to tesamorelin at week 26 and those who did not. For each set of participants, comparisons are made between patients with and without dorsocervical fat deposition at baseline.

Baseline comparisons between groups (patients with and without dorsocervical fat) were made using the Cochran–Mantel–Haenzel statistic, for categorical variables, and an analysis of variance, for continuous variables. To compare changes from baseline to 26 weeks between patients with and without dorsocervical fat groups, analysis of covariance was used, with control for baseline value and study (and lipid-lowering treatment for lipid parameters). Within-group comparisons were done using mixed repeating measure models, with control for study. Mean values ± standard deviation (SD) are reported.

## Results

### Baseline Characteristics

Overall, there were 130 (39%) patients with dorsocervical fat and 207 (61%) patients without dorsocervical fat. Baseline characteristics of the two groups are reported in Table 1. Both groups skewed older, white males with no statistical differences between groups in age, race, or sex. A larger proportion of individuals in those with dorsocervical fat had undetectable viral loads (79.2% vs 73.4%; *P* = 0.028); however, ART regimens (*P* = 0.906) and CD4 counts (648.3 cells/mm^3^ vs 584.1 cells/mm^3^; *P* = 0.08) were similar between the two groups (Table 1). Body composition also differed between the two groups at baseline. Those with dorsocervical fat had higher overall BMIs (30.169 kg/m^2^ vs 27.84 kg/m^2^; *P* < 0.001), higher WC (106.91 cm vs 102.92 cm; *P* < 0.001), and lower VAT:SAT ratio (1.0654 vs 1.4198; *P* = 0.025) than their counterparts without dorsocervical fat (Table 1).

**Table 1.** Baseline characteristics of tesamorelin subjects at week 26, by dorsocervical status prior to receipt of tesamorelin

Variable	With Dorsocervical Fat (*n* = 130)	Without Dorsocervical Fat (*n* = 207)	*P* value
Sex, *n* (%)			0.11
Male	108 (83.1)	184 (88.9)	
Age (years)			0.621
* n*	130	207	
Mean (SD)	47.8 (6.85)	47.4 (7.42)	
Race (Combined), *n* (%)			0.066
Black	19 (14.6)	18 (8.7)	
Other	8 (6.2)	23 (11.1)	
White	103 (79.2)	166 (80.2)	
ART as Baseline, *n* (%)			0.906
NRTI-NNRTI-NO PI	50 (38.5)	75 (36.2)	
NRTI-NNRTI-PI	13 (10.0)	18 (8.7)	
NRTI-PI-NO NNRTI	54 (41.5)	93 (44.9)	
NRTI Alone	5 (3.8)	10 (4.8)	
Other	8 (6.2)	11 (5.3)	
CD4 Cell count (cells/mm^3^)			0.080
* n*	130	206	
Mean (SD)	648.3 (290.63)	584.1 (279.56)	
Viral Load (cp/mL), *n* (%)			0.028
< 50	103 (79.2)	152 (73.4)	
50–400	20 (15.4)	31 (15.0)	
> 400	7 (5.4)	23 (11.1)	
BMI (kg/m^2^)			<0.001
* n*	130	207	
Mean (SD)	30.169 (4.6428)	27.84 (3.4015)	
Presence of Lipoatrophy, *n* (%)			0.063
Yes	102 (78.5)	141 (68.1)	
Waist circumference (cm)			<0.001
* n*	130	207	
Mean (SD)	106.91 (10.906)	102.92 (7.718)	
VAT (cm^2^)			0.913
* n*	130	207	
Mean (SD)	186.592 (85.1752)	190.064 (80.637)	
VAT:SAT			0.025
* n*	126	205	
Mean (SD)	1.0654 (0.988)	1.4198 (1.578)	
IGF-1 level (ng/ml)			0.099
* n*	128	203	
Mean (SD)	149.3 (61.84)	158.4 (62.92)	

Abbreviations: ART- antiretroviral therapy, NRTI- nucleoside reverse transcriptase inhibitors, NNRTI- non-nucleoside reverse transcriptase inhibitors, PI- protease inhibitors, SD- standard deviation, BMI- body mass index, VAT- visceral adipose tissue, SAT- subcutaneous adipose tissue, IGF- insulin-like growth factor.

Importantly, the same proportion of patients considered VAT responders at week 26 were observed in patients with dorsocervical fat (88 out of 130 patients, 68%) and in patients without dorsocervical fat (144 out of 207 patients, 70%). As such, subsequent analyses focused on tesamorelin responders to characterize the impact, if any, of dorsocervical fat on response to tesamorelin. Equivalent analyses were performed in the non-responder group for completion.

### Anthropometric

Abdominal fat parameters were measured at week 26 in patients with and without dorsocervical fat (Table 2 and 3). Among responder patients, there were VAT reductions in both groups with a 50.00 cm^2^ decrease in the group with dorsocervical fat (*P* < 0.001) and a 50.23 cm^2^ decrease in the group without dorsocervical fat (*P* < 0.001). While this subanalysis is limited to responder individuals, who by definition have an 8% reduction in VAT, it is important to understand whether the magnitude of this response differs by dorsocervical fat status. Importantly, there was no difference between the two groups in terms of change from baseline (*P* = 0.66). In line with this finding, WC decreased in both groups as well. Responders with dorsocervical fat had a 3.62-cm reduction (*P* < 0.001) and those without had a 4.58-cm reduction (*P* < 0.001). These reductions did not differ between groups (*P* = 0.093).

**Table 2.** Change in abdominal adiposity, Insulin-like growth factor-1 levels, and metabolic parameters between baseline and week 26 among tesamorelin responder patients, by dorsocervical status

	Patients with Dorsocervical Fat (*n* = 88)				Patients without Dorsocervical Fat (*n* = 144)				
Variable	Baseline	Week 26	Change	*P*-value within group	Baseline	Week 26	Change	*P*-value within group	*P*-value between group
VAT (cm^2^)									0.657
*n*	88	88	88		144	144	144		
Mean (SD)	189.68 (87.04)	139.67 (67.95)	-50.01 (33.13)	<0.001	184.88 (78.59)	134.65 (69.21)	-50.23 (33.95)	<0.001	
SAT (cm^2^)									0.009
*n*	85	85	85		142	142	142		
Mean (SD)	246.77 (126.95)	243.22 (119.41)	-3.56 (42.02)	0.44	204.00 (107.68)	192.72 (101.42)	-11.28 (31.11)	<0.001	
Waist Circumference (cm)									0.093
*n*	88	88	88		143	143	143		
Mean (SD)	105.99 (9.95)	102.37 (10.18)	-3.62 (6.35)	<0.001	102.35 (7.41)	97.77 (9.12)	-4.58 (5.24)	<0.001	
Trunk Fat (kg)									0.301
*n*	83	83	83		143	143	143		
MeanMean (SD)	15.58 (4.73)	13.90 (4.74)	-1.68 (2.06)	<0.001	13.71 (4.54)	11.87 (4.81)	-1.84 (2.03)	<0.001	
Fat in Limbs (kg)									0.008
*n*	83	83	83		143	143	143		
Mean (SD)	6.99 (3.74)	6.91 (3.68)	-0.086 (1.07)	0.47	6.48 (3.82)	6.06 (3.53)	-0.41 (0.98)	<0.001	
Lean Mass (kg)									0.144
*n*	83	83	83		143	143	143		
Mean (SD)	62.97 (9.64)	64.25 (10.24)	1.29 (2.08)	<0.001	61.75 (9.55)	63.54 (9.72)	1.79 (2.59)	<0.001	
IGF-1									0.073
*n*	85	85	85		142	142	142		
Mean (SD)	148.70 (61.55)	298.60 (135.92)	149.90 (109.30)	<0.001	154.10 (60.64)	281.40 (118.92)	127.40 (103.54)	<0.001	
Adiponectin (ug/mL)									0.487
*n*	53	53	53		69	69	69		
Mean (SD)	4.80 (2.86)	5.90 (3.11)	1.10 (1.32)	<0.001	6.04 (5.38)	7.20 (7.30)	0.90 (3.89)	0.062	
Total Cholesterol (mmol/L)									0.041
*n*	85	85	85		143	143	143		
Mean (SD)	4.98 (1.05)	4.91 (0.96)	-0.067 (0.73)	0.401	5.09 (1.23)	4.82 (1.03)	-0.27 (0.94)	<0.001	
Triglycerides (mmol/L)									0.278
*n*	88	88	88		144	144	144		
Mean (SD)	2.770 (2.3613)	2.236 (1.5389)	-0.534 (1.7289)	0.005	2.669 (2.1513)	2.070 1.2309)	-0.600 (1.622)	<0.001	
HbA1C (%)									0.376
*n*	81	81	81		137	137	137		
Mean (SD)	5.34 (0.43)	5.40 (0.50)	0.063 (0.31)	0.070	5.21 (0.51)	5.28 (0.58)	0.075 (0.36)	0.015	
CD4 cell count (cells/mm^3^)									0.439
*n*	86	86	86		143	143	143		
Mean (SD)	658.72 (299.78)	629.13 (256.88)	-29.59 (130.47)	0.038	577.90 (280.76)	581.76 (250.57)	3.87 (153.93)	0.76	

Abbreviations: VAT- visceral adipose tissue, SAT- subcutaneous adipose tissue, IGF-1- insulin like growth factor 1, SD- standard deviation

**Table 3.** Change in abdominal adiposity, Insulin-like growth factor-1 levels, and metabolic parameters between Baseline and week 26 among Tesamorelin non-responder patients, by Dorsocervical status

	*Patients with Dorsocervical Fat (n = 42)*				*Patients without Dorsocervical Fat (n = 63)*				
Variable	Baseline	Week 26	Change	*P*-value within group	Baseline	Week 26	Change	*P*-value within group	*P*-value between group
VAT (cm^2^)									0.273
* n*	42	42	42		63	63	63		
Mean (SD)	180.13 (81.77)	198.81 (91.70)	18.68 (29.46)	<0.001	201.92 (84.59)	216.83 (87.89)	14.91 (26.81)	<0.001	
SAT (cm^2^)									0.504
* n*	40	40	40		61	61	61		
Mean (SD)	257.13 (126.65)	274.49 (127.36)	17.47 (29.14)	<0.001	214.29 (124.61)	222.18 (122.48)	7.90 (34.94)	0.083	
Waist Circumference (cm)									0.187
* n*	42	42	42		59	59	59		
Mean (SD)	108.85 (12.60)	107.70 (13.49)	-1.15 (5.31)	0.17	104.27 (8.32)	104.37 (9.76)	0.09 (3.67)	0.84	
Trunk Fat (kg)									0.567
* n*	37	37	37		59	59	59		
Mean (SD)	16.92 (6.20)	17.50 (6.44)	0.58 (1.69)	0.04	14.79 (5.03)	15.34 (5.09)	0.55 (1.81)	0.024	
Fat in Limbs (kg)									0.390
* n*	37	37	37		59	59	59		
Mean (SD)	7.98 (4.55)	8.53 (4.57)	0.55 (0.98)	0.002	6.625 (4.09)	7.04 (4.20)	0.42 (0.73)	<0.001	
Lean Mass (kg)									0.416
* n*	37	37	37		59	59	59		
Mean (SD)	62.66 (10.89)	64.17 (11.16)	1.52 (2.69)	0.002	64.10 (10.69)	65.51 (10.66)	1.07 (2.43)	0.001	
IGF-1									0.055
* n*	42	42	42		60	60	60		
Mean (SD)	153.10 (61.64)	223.1 (90.81)	70.0 (83.29)	<0.001	168.80 (67.89)	246.30 (104.95)	95.60 (115.82)	<0.001	
Adiponectin (ug/mL)									0.417
* n*	25	25	25		21	21	21		
Mean (SD)	4.60 (2.47)	4.60 (2.49)	0.0 (1.56)	0.949	5.40 (4.14)	4.80 (3.33)	-0.6 (2.08)	0.174	
Total Cholesterol (mmol/L)									0.090
* n*	42	42	42		61	61	61		
Mean (SD)	5.037 (1.03)	5.22 (1.17)	0.185 (0.93)	0.207	4.84 (1.02)	4.89 (1.11)	0.05 (0.97)	0.708	
Triglycerides									0.932
* n*	42	42	42		63	63	63		
Mean (SD)	2.732 (2.1063)	2.495 (2.0919)	-0.238 (1.2905)	0.240	2.430 (1.4314)	2.378 (1.3638)	-0.051 (1.1193)	0.717	
HbA1C (%)									0.467
* n*	41	41	41		59	59	59		
Mean (SD)	5.32 (0.55)	5.51 (0.69)	0.19 (0.43)	0.007	5.21 (0.49)	5.59 (0.73)	0.31 (0.40)	0.467	
CD4 cell count (cells/mm3)									0.237
* n*	41	41	41		62	62	62		
Mean (SD)	630.22 (282.53)	607.10 (273.05)	-23.12 (205.87)	0.476	597.24 (280.73)	611.86 (304.07)	14.61 (150.86)	0.449	

Abbreviations: VAT- visceral adipose tissue, SAT- subcutaneous adipose tissue, IGF-1- insulin-like growth factor 1, SD- standard deviation.

In contrast, among nonresponder patients, there was a VAT increase in both groups with a 18.68 cm^2^ increase in the group with dorsocervical fat (*P* < 0.001) and a 14.91cm^2^ increase in the group without dorsocervical fat (*P* < 0.001). This increase in VAT from baseline did not differ between the two dorsocervical groups (*P* = 0.27). Furthermore, there was no statistically significant change in WC in either nonresponder group.

Among VAT responders, both groups had statistically significant decreases in trunk fat and increases in lean mass (Table 2). These changes did not differ by dorsocervical fat status. VAT responder patients without dorsocervical fat did, however, have additional significant decrease in subcutaneous adipose tissue (SAT) and limb fat that their counterparts did not (Table 2).

Nonresponders, in contrast, had increases in all four parameters: trunk fat, lean mass, SAT, and limb fat (Table 3). While these changes did not differ between groups with and without dorsocervical fat, the SAT increase was only statistically significant in individuals with dorsocervical fat (Table 3).

### Metabolic

Metabolic parameters were also measured at week 26 and changes are reported for both responder and nonresponder groups with and without dorsocervical fat (Tables 2 and 3). Responders and nonresponders both had increases in IGF-1. This did not differ by dorsocervical fat status in either group. Adiponectin increased in responders with dorsocervical fat, but not in responders without dorsocervical fat. It did not change in nonresponders regardless of dorsocervical fat. Responders had a decrease in triglycerides which did not differ between those with and without dorsocervical fat. There was also a decrease in total cholesterol among responders with dorsocervical fat. Nonresponders did not have changes in any lipid parameters. HbA1c increased in responders without dorsocervical fat, but not those with dorsocervical fat. HbA1c increased in both nonresponder groups; however, it did not differ between those with and without dorsocervical fat. Lastly, there was a statistically significant decrease in CD4 counts in the responder group with dorsocervical fat, which was not observed in the nonresponder group.

## Discussion

Tesamorelin has previously been shown to decrease VAT in a phase III clinical study of tesamorelin compared to placebo [12]. The study showed that patients who received tesamorelin had significant reductions in VAT at week 26 and maintained that reduction at week 52. The medication was well tolerated without significant changes to glucose-based measurements [12]. Previous post hoc analyses were performed to characterize tesamorelin responders and identify predictors of response [14,15]. They found that patients who had baseline metabolic syndrome, elevated triglyceride levels or were of white race were more likely to exhibit a decrease in VAT after 6 months of tesamorelin therapy [14]. This study, however, did not evaluate the impact of ectopic fat, a common component of HIV-associated lipodystrophy, on response to tesamorelin.

Our current data demonstrate that tesamorelin is effective at reducing VAT in patients regardless of dorsocervical fat deposition. Indeed, the presence of dorsocervical fat was associated with very few differences in central anthropometric measurements. Interestingly, only responders without dorsocervical fat had additional reductions in SAT and limb fat at week 26 which may be due to baseline differences in fat burden or could reflect differences in adipose function in individuals with dorsocervical fat [16,17]. Metabolic measurements were similarly equivalent between those with and without dorsocervical fat. While there were some differences in HIV-related parameters, these did not consistently differ by dorsocervical status at baseline and week 26. Furthermore, neither CD4 count nor viral load has been previously shown to differ by tesamorelin response [14]. This has important clinical implications as tesamorelin is the only FDA-approved treatment for the reduction of VAT and data were lacking as to whether persons who also had excessive dorsocervical fat would still benefit from its use in VAT reduction [11]. The current study had limitations due to the focus on VAT reduction among persons who received tesamorelin and exclusion of those who received placebo. Due to the cross-over study design, half of all tesamorelin recipients were switched to placebo at week 26. This resulted in a decrease in participants that met the responder definition from week 26 (n = 232) to week 52 (n = 110). For this reason, we did not evaluate results at 52 weeks so as not to introduce additional bias.

In addition, this study does not include the impact of lipoatrophy on tesamorelin response. As such, there remain questions about VAT deposition and treatment in patients with combined lipoatrophy and lipohypertrophy. Interestingly, a defect in peripheral adipocytes leads to greater availability of fatty acids in circulation, which then deposit in available sites, including VAT [18]. Thereby fat may be unable to develop in areas of prior lipoatrophy.

Regardless of the manifestation, lipodystrophy is concerning to patients. Tesamorelin can be considered in patients to reduce VAT irrespective of dorsocervical lipohypertrophy.
